# RNA Interference in *Schistosoma mansoni* Schistosomula: Selectivity, Sensitivity and Operation for Larger-Scale Screening

**DOI:** 10.1371/journal.pntd.0000850

**Published:** 2010-10-19

**Authors:** Saša Štefanić, Jan Dvořák, Martin Horn, Simon Braschi, Daniel Sojka, Debbie S. Ruelas, Brian Suzuki, Kee-Chong Lim, Stephanie D. Hopkins, James H. McKerrow, Conor R. Caffrey

**Affiliations:** 1 Sandler Center for Drug Discovery, California Institute for Quantitative Biosciences (QB3), University of California San Francisco, San Francisco, California, United States of America; 2 Institute of Organic Chemistry and Biochemistry, Academy of Sciences of the Czech Republic, Praha, Czech Republic; 3 Institute of Parasitology, Biology Centre, Academy of Sciences of the Czech Republic, České Budějovice, Czech Republic; University of Queensland, Australia

## Abstract

**Background:**

The possible emergence of resistance to the only available drug for schistosomiasis spurs drug discovery that has been recently incentivized by the availability of improved transcriptome and genome sequence information. Transient RNAi has emerged as a straightforward and important technique to interrogate that information through decreased or loss of gene function and identify potential drug targets. To date, RNAi studies in schistosome stages infecting humans have focused on single (or up to 3) genes of interest. Therefore, in the context of standardizing larger RNAi screens, data are limited on the extent of possible off-targeting effects, gene-to-gene variability in RNAi efficiency and the operational capabilities and limits of RNAi.

**Methodology/Principal Findings:**

We investigated *in vitro* the sensitivity and selectivity of RNAi using double-stranded (ds)RNA (approximately 500 bp) designed to target 11 *Schistosoma mansoni* genes that are expressed in different tissues; the gut, tegument and otherwise. Among the genes investigated were 5 that had been previously predicted to be essential for parasite survival. We employed mechanically transformed schistosomula that are relevant to parasitism in humans, amenable to screen automation and easier to obtain in greater numbers than adult parasites. The operational parameters investigated included defined culture media for optimal parasite maintenance, transfection strategy, time- and dose- dependency of RNAi, and dosing limits. Of 7 defined culture media tested, Basch Medium 169 was optimal for parasite maintenance. RNAi was best achieved by co-incubating parasites and dsRNA (standardized to 30 µg/ml for 6 days); electroporation provided no added benefit. RNAi, including interference of more than one transcript, was selective to the gene target(s) within the pools of transcripts representative of each tissue. Concentrations of dsRNA above 90 µg/ml were directly toxic. RNAi efficiency was transcript-dependent (from 40 to >75% knockdown relative to controls) and this may have contributed to the lack of obvious phenotypes observed, even after prolonged incubations of 3 weeks. Within minutes of their mechanical preparation from cercariae, schistosomula accumulated fluorescent macromolecules in the gut indicating that the gut is an important route through which RNAi is expedited in the developing parasite.

**Conclusions:**

Transient RNAi operates gene-selectively in *S. mansoni* newly transformed schistosomula yet the sensitivity of individual gene targets varies. These findings and the operational parameters defined will facilitate larger RNAi screens.

## Author Summary

RNA interference (RNAi) is a technique to selectively suppress mRNA of individual genes and, consequently, their cognate proteins. RNAi using double-stranded (ds) RNA has been used to interrogate the function of mainly single genes in the flatworm, *Schistosoma mansoni*, one of a number of schistosome species causing schistosomiasis. In consideration of large-scale screens to identify candidate drug targets, we examined the selectivity and sensitivity (the degree of suppression) of RNAi for 11 genes produced in different tissues of the parasite: the gut, tegument (surface) and otherwise. We used the schistosomulum stage prepared from infective cercariae larvae which are accessible in large numbers and adaptable to automated screening platforms. We found that RNAi suppresses transcripts selectively, however, the sensitivity of suppression varies (40%–>75%). No obvious changes in the parasite occurred post-RNAi, including after targeting the mRNA of genes that had been computationally predicted to be essential for survival. Additionally, we defined operational parameters to facilitate large-scale RNAi, including choice of culture medium, transfection strategy to deliver dsRNA, dose- and time-dependency, and dosing limits. Finally, using fluorescent probes, we show that the developing gut allows rapid entrance of dsRNA into the parasite to initiate RNAi.

## Introduction

Schistosomiasis is one of a number of ‘neglected tropical diseases’ (NTDs) associated with extreme poverty in the world's ‘bottom billion’ people [1]. Caused by a parasitic flatworm, the disease can be fatal, but is better known for its chronicity of infection (e.g., [2]). Clinical manifestations of chronic infection include malaise, anemia and wasting, and poor cognitive performance in children [3], [4], [5], [6], [7]. Infection with *Schistosoma haematobium* is associated with an increased risk of bladder cancer [8], [9] and due to the compromised integrity of the lower female genital tract, with HIV infection [1], [9], [10].

Because treatment and control of schistosomiasis has come to rely on a single drug, praziquantel (PZQ; [11], [12], [13]), concern remains over the possible emergence and establishment of clinically relevant drug resistance [14]. There are reports of decreased, if sometimes, transient [15], susceptibility by field-isolates of parasite to PZQ [16]. The risk of resistance to PZQ [14], [17] is all the more relevant given the impetus to increase access to this and other anthelmintics via integrated mass administration programs [18], [19], [20], [21], [22]. Thus, it remains a priority to identify and develop alternative chemo- and immuno-therapeutic interventions for schistosomiasis, and progress is being made in both these areas [23], [24], [25], [26], [27].

Key initial tasks for drug development are the identification and validation of gene products for which modulation by chemical and/or genetic means translates to impaired cell/organism survival. For schistosomes, comprehensive transcriptome [28], [29], [30], [31], [32], [33], [34], [35], [36] and genome sequence information [37], [38], [39] is now accessible through interrogable databases [37], [40], [41], [42]. These databases have facilitated new strategies, e.g., [43], [44], [45], with which potential gene targets have been identified, e.g., [46]. Coincident with these developments is the continued focus to improve a number of reverse genetic tools to define gene function [47], [48], [49], [50]. Since the first reports in 2003 [51], [52], transient RNA interference (RNAi) with either long or short-interfering (si) double-stranded (ds)RNA has proven a straightforward and important tool to study loss of or decreased function for selected genes of interest, including proteases [53], [54], [55], [56],kinases [57], TGF-beta receptor [58], aquaporin [59], glucose transporters [60], tetraspanins [61] and redox-associated enzymes [62], [63]. In some of these studies obvious phenotypes that affected parasite vitality and/or survival were obtained. This evidence for RNAi in schistosomes is underpinned by *in silico* and experimental data to suggest that the necessary RNAi molecular machinery is present [33], [64], [65], [66]. Accordingly, larger scale RNAi screening campaigns of schistosomes can be now envisaged.

To date, RNAi studies with schistosomes relevant to infection in humans have focused on single (or up to 3; [55], [65]) genes of interest using different developmental stages and experimental conditions. Thus, the studies only partially contribute to standardizing a set of conditions that are applicable to larger scale investigations of gene function. Furthermore, the degree to which complicating factors such as individual gene susceptibility to RNAi and ‘off-targeting’ (whereby dsRNA can direct the RNAi machinery to target transcripts other than that intended) occur is unknown. The latter phenomenon can confound interpretations of loss-of-function [67], [68], [69], [70], [71], [72], especially when phenotypes are generated [73]. Both issues were highlighted in a recent RNAi phenotypic screen of 32 genes in the mother sporocyst stage of *Schistosoma mansoni* that parasitizes the snail vector [74], [75].

As a pre-requisite to larger RNAi-based screens of schistosome stages infecting humans, we investigated the issues of target selectivity and sensitivity of RNAi for 11 gene transcripts expressed in different tissues of *S. mansoni*, including genes predicted *in silico* as being essential to parasite survival [46]. We focused on mechanically transformed schistosomula, which, compared to adult schistosomes, are easier to procure in their hundreds of thousands from snails and adaptable to automated higher throughput screening technologies [76]. Schistosomula also offer the opportunity to identify genes of potential value as chemo- or immuno-therapeutic targets prior to the onset of disease pathology and morbidity associated with egg-laying adults. At the same time, we examined a number of operational parameters (optimal medium for parasite maintenance, transfection strategy and time-, and dose-dependencies of RNAi) for which refinement would better facilitate larger-scale loss-of-function screens.

## Materials and Methods

### Ethics Statement

Maintenance of and experiments with vertebrate animals were carried out in accordance with protocols approved by the Institutional Animal Care and Use Committee (IACUC) at UCSF.

### *S. mansoni* life-cycle and preparation of schistosomula

A Puerto Rican isolate of *S. mansoni* is maintained in the laboratory using the intermediate snail host, *Biomphalaria glabrata* and the Golden Syrian hamster, *Mesocricetus auratus*, (infected at 5–6 weeks old; Simonsen) as the definitive host [76]. Methods for the preparation of newly transformed schistosomula (hereafter termed schistosomula) are described previously [76]. Essentially, the procedure involves cleaning cercariae from snail water and mechanically shearing cercarial tails from heads [77] (the nascent schistosomula) in ice-chilled Basch Incomplete Medium 169 [78] (custom synthesized at the UCSF Cell Culture Facility; containing 100 U/ml penicillin and 100 µg/ml streptomycin, but without FBS). Schistosomula are collected and washed three times in the same medium and kept on ice for a maximum of 1 h prior to dsRNA treatment.

### Media evaluation test

To compare the effects of different defined culture media on parasite vitality, 200–300 schistosomula were incubated in 96-well flat-bottom plates (Corning) containing 200 µL medium, 5% FBS and 100 U/ml penicillin and 100 µg/ml streptomycin for up to 7 days at 5% CO_2_ and 37°C. Media tested were: Basch Medium 169; DMEM (high glucose; UCSF cell culture facility code CCFAA005); F-12 (CCFAB002); Liebovitz's L-15 Medium without NaHCO_3_ (CCFDY003); M199 with Earle's Salts (CCFAX001); RPMI 1640 (containing 2mM glutamine (CCFAE001) and supplemented with 10 mM HEPES), and Schneider's Drosophila Medium (INVZR512). Phenotypic alterations in translucency, shape and motility [76] were assessed every second day. Still and time-lapse image recordings were made on the seventh day.

### Transcript targets

We selected 11 genes encoding diverse proteins and putatively expressed in different tissues (gut, tegument and otherwise) in order to assess any variability in the ability to suppress gene transcription. For gut genes, we chose 3 well-characterized *S. mansoni* proteases; cathepsin B1 (using a dsRNA targeting both isoforms - CB1.1 (AJ506157) and CB1.2, (AJ506158), cathepsin C (CC, Z32531) and cathepsin D (CD, U60995) [54], [79]. For the tegument, 3 genes were chosen; CB2 (AJ312106), annexin (XP_002578945.1) and Sm29 (XP_002578407.1). The respective proteins have been bio/immuno-chemically localized to the parasite surface [80], [81], [82] although CB2 is also localized in the parenchyma [82]. Annexin is also a protein common to most eukaryote plasma membranes [83] and may, therefore, be more generally distributed in the parasite. The remaining 5 targets are considered to be more generally distributed and have been predicted to be essential in *S. mansoni* in a recent comparative genomics analysis [46]: glycogen synthase kinase-3 (GSK3; XP_002572305.1), methionine aminopeptidase (MetAP; XP_002572573.1), protein phosphatase-2a (PP-2a; XP_002574514.1), neuroendocrine convertase (NEC; XP_002569775.1) and n-myristoyl transferase (nMT; XP_002571399.1). Table S1 describes the primers used for dsRNA synthesis (the T7 DNA polymerase binding motif is shown in bold characters) and the quantitative real-time (qRT)-PCR primers used to measure RNAi. For dsRNA design, the nucleotide sequence encoding the open reading frame of each gene target was examined using the nucleotide BLAST service at NCBI (constrained to the *Schistosoma* taxid ID of 6181). Sequence areas displaying homology (especially for identical stretches of 10 nucleotides or more) with other *Schistosoma* genes were avoided in order to decrease the risk of off-targeting.

### Synthesis of dsRNA and Cy5-labeled dsRNA

PCR products of approximately 500 bp in size were amplified from cDNA prepared from 6 day-old *in vitro* cultivated *S. mansoni* schistosomula cDNA using gene-specific primers for the targeted genes (Table S1). The expected size of the amplicon was measured by electrophoresis in 1% agarose gels and identity confirmed by DNA sequencing. Amplicons were then used as templates for two more PCR reactions using either a sense or antisense primer containing a 5′-positioned T7 polymerase promoter sequence. After gel purification, each of the two PCR products was separately reverse transcribed *in vitro* using the T7 RiboMAX express RNAi system (Promega) and the resulting single-stranded RNA pooled to generate dsRNA that was then treated with RNase A and DNase. The manufacturer's instructions were applied apart from scaling the reverse transcription reaction to 100 µl and allowing it to proceed overnight at 37°C with a final hour at 42°C. The dsRNA was precipitated using 1 volume of 10% 3 M sodium acetate (pH 5.2) in 100% isopropanol, and pelleted at 12 000 *g* for 15 min at 4°C. The pellet was washed in 70% ethanol, air-dried and resuspended in water. The concentration of dsRNA was measured in an ND-1000 Spectrophotometer (Nanodrop Technologies) and adjusted to 3.0 mg/ml. The size and integrity of the resulting dsRNA was confirmed by electrophoresis in 1% agarose gels and stored at −80°C until use.

For synthesis of Cy5-labeled dsRNA to CC, CB1 and the *Discosoma* sp. fluorescent protein, mCherry ([84]; GenBank AY678264), an aliquot of dsRNA (40 µg) was labeled using the *Label* IT Nucleic Acid Labeling Reagent (Mirus) according to the manufacturer's instructions. Labeling efficiency was monitored using an ND-1000 Spectrophotometer.

### Treatment of schistosomula with dsRNA

Schistosomula were divided into groups of approximately 400 and incubated in 24-well plates containing 1 ml Complete Medium 169 (plus 5% FBS) and dsRNA (30 µg unless otherwise stated) targeting either schistosome transcripts or mCherry that was employed as a schistosome-unspecific control. Incubations were continued for 6 days at 37°C and 5% CO_2_. To isolate for the effect of electroporation on RNAi efficiency, the following additional incubations were set up. Groups of 400 schistosomula were transferred to 4 mm electroporation cuvets (Bio-Rad Laboratories) in Basch Incomplete Medium 169. DsRNA (30 µg) was then added (usually as a 10 µL aliquot) in Incomplete Medium 169 to give a final volume of 100 µl. Electroporation was carried at 125 V for 20 ms [53] using a Bio-Rad GenePulser Xcell. Worms were then suspended in 1 ml Complete Medium 169 and incubations continued for 6 days. A second group of worms was electroporated as above, but following electroporation, worms were washed 10 times in Incomplete Medium 169 to remove as much dsRNA as possible and then incubated at 37°C. A third group was electroporated in the absence of dsRNA, and immediately thereafter dsRNA was added for incubation at 37°C.

### RNA isolation from schistosomula after dsRNA treatment and conversion to cDNA

After 6 days of cultivation, schistosomula were collected, washed 3 times in 1.5 ml PBS and resuspended in 50 µl of Trizol reagent (Invitrogen). Samples were flash frozen in liquid nitrogen and stored at −80°C. During thawing, samples were homogenized with Kontes disposable pellet pestles and microtubes (VWR). Sample volumes were adjusted to 500 µl with Trizol reagent and the temperature adjusted to 65°C for 5 min to improve tissue lysis. To separate nucleoprotein complexes, samples were further incubated at room temperature for 15–20 minutes. Steps to continue the isolation of total RNA with Trizol were performed up until phase separation in chloroform. At this point, the aqueous phase was transferred to a new 1.5 ml microfuge tube containing an equal volume of 75% ethanol. RNA was further purified using the RNeasy Mini Kit (Qiagen) according to the supplied instructions. The concentration of RNA was determined using an ND-1000 Spectrophotometer. Single-stranded cDNA was synthesized from total RNA using SuperScript III reverse transcriptase (Invitrogen) and an oligo d(T)_18_ reverse primer according to the manufacturer's protocol. The resulting cDNA was purified using a DNA Clean & Concentrator-5 (Zymo Research Corporation) and stored in water at −80°C.

### Primer design and optimization, and qRT-PCR analyses

Primers were designed using the Primer 3 software (http://frodo.wi.mit.edu/, [85]) employing a target product size of 150–200 bp and a primer melting temperature of 55°C. Primer design excluded outputs containing stretches of four or more of identical nucleotides that might interfere with binding specificity. Primers were designed to bind outside of the region targeted by the dsRNA to avoid the possibility of amplifying carryover PCR product used as a template in dsRNA synthesis reaction. The specificity of each primer sequence was confirmed by BLAST analysis. Primers were purchased from Integrated DNA Technologies at the 25 nM scale and without additional purification procedures. The size of PCR amplicon generated by each primer pair was visually inspected by 1% agarose gel electrophoresis to ensure that the intended sized product was in fact amplified. The amplification efficiency of each qRT-PCR primer set was evaluated in triplicate over at least three and five dilutions of primers and cDNA template, respectively. The cycle threshold (C_T_) values arising were plotted as a function of the dilution of cDNA [86]. Optimal efficiencies generated curves with slopes of between −3.5 and −3.2 (100% efficiency is represented by a gradient of −3.32). R^2^ coefficients of ≥0.98 indicated high reproducibility for the triplicate reactions. Primer sets were also checked for their dissociation (melting) curves using the appropriate setting of the MxPro QPCR software package (see below). Only those primers displaying optimal efficiencies and generating single dissociation peaks (the latter demonstrative of a specific reaction without, for example, primer dimerization) [87] were considered further. Our experience has been that, for each transcript target, two to four primer sets needed to be tested in order to identify an optimal pair.

Quantitative RT-PCR was carried out in an MX 3005P Real-Time PCR cycler (Stratagene, Agilent Technologies, Inc.) using the LightCycler 480 SYBR Green I Master mix (Roche Diagnostics). *S. mansoni* cytochrome C oxidase I (GenBank AF216698, [88]) served as the sample normalizing gene transcript [89]. This transcript is highly and constitutively expressed in a number of *S. mansoni* life-cycle stages [30]. Controls for genomic DNA contamination (minus reverse transcriptase) and reagent purity (water control) were included for each sample. Reactions were carried out in a final volume of 25 µl in 96 well plates (Stratagene). The amplification profile consisted of an initial hot start (95°C for 10 min), followed by 40 cycles comprising 95°C for 30 s, 55°C for 60 s and 72°C for 60 s, and ended with a single cycle of 95°C for 60 s, 55°C for 30 s and 95°C for 30 s. PCR reactions were performed in duplicate for each cDNA sample. Also, at least one additional biological replicate, i.e., with cDNA arising from a separate dsRNA application, was performed. To ensure precision, the entire qPCR experiment was repeated when C_T_ values of duplicate reactions differed by 0.5 or more (indicative of pipeting error). Using the MxPro QPCR Software version 4.01 (Stratagene), the 2^−ΔΔC^_T_ method [90] was applied to measure transcript levels following dsRNA treatment. Transcript levels were expressed as a percentage of those following exposure to schistosome-unspecific mCherry dsRNA.

### Irreversible active site labeling of SmCC

To verify RNAi of SmCC at the protein level, the fluorescent and selective cathepsin C affinity probe bodipy-labeled FY01 [91] was incubated with protein extract from schistosomula exposed to SmCC-dsRNA. Extracts from worms incubated with mCherry-dsRNA served as a control. Soluble protein extracts were prepared by sonication in 50 mM citrate, 100 mM phosphate, pH 5.0 over an ice bath. After centrifugation at 8 000 *g* and 4°C for 5 min, supernatants were collected. An aliquot (2 µg protein) was incubated for 1 h at 37°C with 1 µM bodipy-labeled FY01 in 50 mM sodium acetate pH 5.5 containing 2.5 mM DTT, 1 mM EDTA and 1 µM each of the respective aspartic and cathepsin B (cysteine protease) inhibitors, pepstatin A and CA-074 (N-[L-3-trans-propylcarbamoyloxirane- 2-carbonyl]-Ile-Pro-OH [92]; Bachem). The labeling reaction was stopped by heating to 70°C for 15 min in reducing Laemmli sample buffer. The reaction mixture was resolved by SDS-PAGE through 15% polyacrylamide gels and visualized in a Typhoon 8600 Imager (GE Healthcare) using excitation at 532 nm (green laser) and a 580 nm (BP30 nm) emission filter.

SmCC activity was also detected by hydrolysis of the cathepsin C fluorogenic substrate, Gly-Arg-AMC, as described previously [93], in the presence and absence of 10 µM of the following protease inhibitors (Bachem): the general Clan CA cysteine protease inhibitor, K11777 (N-methyl-piperazine-Phe-homoPhe-vinyl sulfone phenyl [94]); the cathepsin B-selective inhibitor, CA-074; and the cathepsin L-preferential inhibitor, Z-Phe-Phe-DMK (benzyloxy carbonyl-Phe-Phe-diazomethane [95]). Assays were performed in triplicate.

### Preparation of recombinant mCherry fluorescent protein

The mushroom anemone (*Discosoma* sp.) red fluorescent protein, mCherry [84], was recombinantly expressed in TOP10 chemically competent *E. coli* (Invitrogen). Bacteria that had been transformed with the mCherry open reading frame as part of a pUC18 plasmid backbone were grown overnight at 37°C in 500 ml LB medium containing 100 µg/ml of ampicillin and 20 mM glucose. After centrifugation at 3 000 *g* for 10 min, bacteria were incubated for 4 h in fresh LB medium containing 1 mM IPTG (isopropyl-β-D-thio-galactoside) and harvested by centrifugation for 10 min at 4 000 *g*. The pellet was re-suspended in 50 ml 100 mM phosphate buffer, pH 8.0, and sonicated using a Branson Sonifier 250 adjusted to a 60% duty cycle and an output control of 6. The material was centrifuged at 10 000 *g* for 15 min and the supernatant, containing recombinant mCherry, was collected. Heat denaturation in a water bath at 80°C for 40 min followed by centrifugation at 10 000 *g* for 15 min was employed to separate bacterial proteins from the heat-stable mCherry protein in the supernatant. The supernatant was decanted and concentrated over an Amicon Ultra-50 10K (Millipore) filter to a final volume of 5 ml. The sample was size fractionated over a Superdex 200 column (GE- Healthcare) pre-equilibrated with 100 mM phosphate buffer, pH 8.0. Eluted fractions containing visible red mCherry protein (4 mg/ml) were pooled and stored at −20°C.

### Uptake of exogenous material by schistosomula *in vitro*

Schistosomula were co-incubated in 1 ml Complete Medium 169 with either 30 µg Cy5-labeled SmCB1- or mCherry- dsRNA, 200 µg recombinant mCherry protein or 2 µL washed and packed human erythrocytes. Uptake was monitored as a function of time, usually every half hour out to 4 h, at 20 h and thereafter, every day out to 4 days. For Cy5-labeled dsRNA, worms were washed 4 times in Incomplete Medium 169 before imaging with a laser scanning microscope (LSM 510 META; Carl Zeiss MicroImaging, Inc.). For mCherry protein, fluorescence was visualized without washing using an Axiovert 40CFL inverted microscope. Bright field images of parasites in the presence of erythrocytes were acquired using an Axiovert 40C inverted microscope.

## Results

### Of seven defined culture media tested, Basch Medium 169 optimally maintains schistosomula

**Figure 1:**
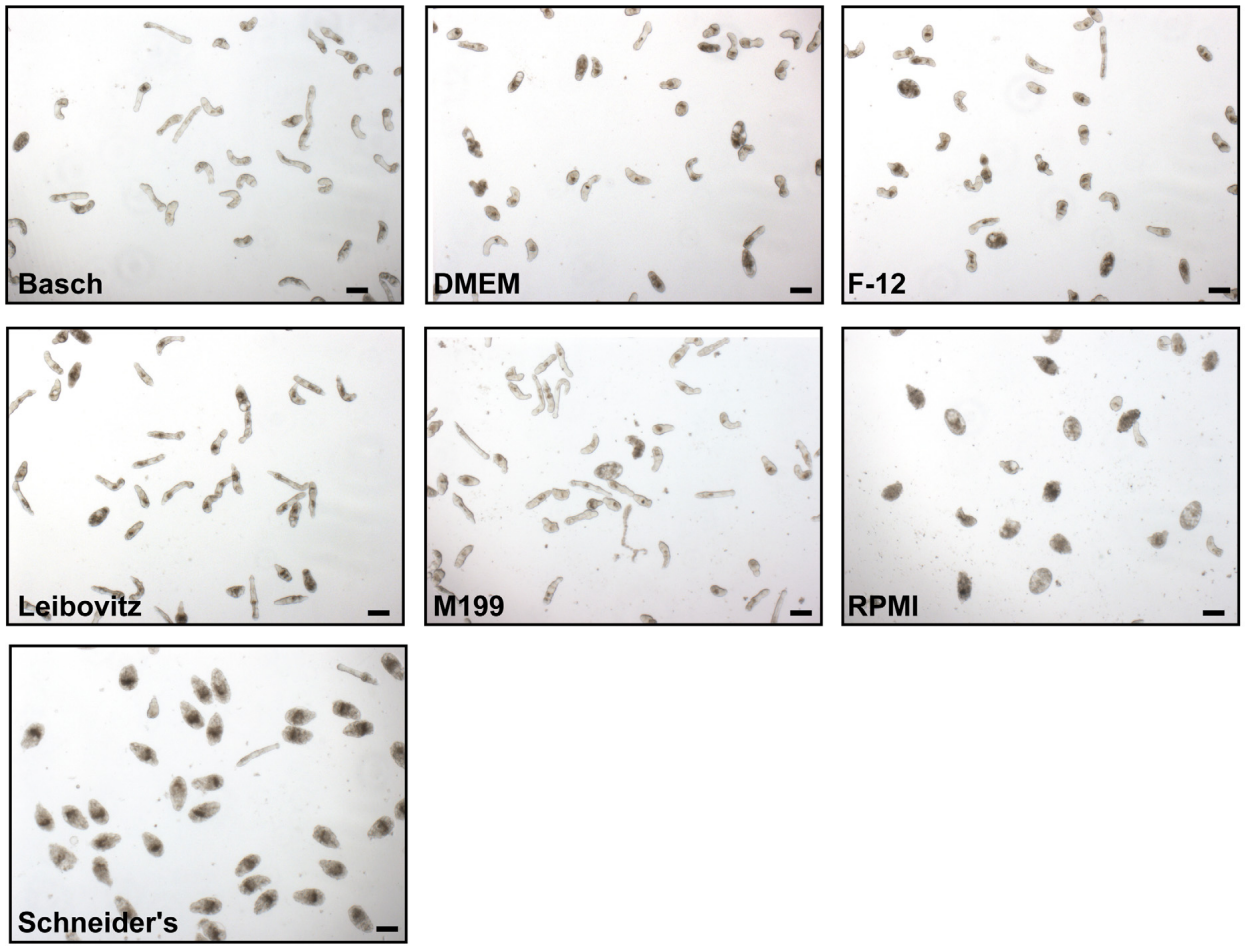
Manifestation of phenotypes in newly transformed *S. mansoni* schistosomula by various defined culture media. Over the course of one week's incubation, parasites in Basch Medium 169 [78] appear robust and uniform in shape and appearance. In contrast, DMEM (high glucose), F-12, Liebowitz and M199 media generated different degrees of distorted parasites including rounding and darkening that was often accompanied by slowed and less dynamic motility. For RPMI and Schneider's medium, parasite rounding, degeneration and death were noted by day 2 of the incubation with >80% parasite mortality by day 7. Images were captured using a Zeiss Axiovert 40 C inverted microscope (10× objective) and a Zeiss AxioCam MRc digital camera controlled by AxioVision 40 version 4.5.0.0 software. These images are best viewed in conjunction with Videos S1 through S7 (taken at the same magnification) to include the effects of media on parasite motility. A second experiment with the same media yielded similar results. Scale bars represent 100 µm.

Prior to RNAi experiments with newly transformed schistosomula, we wished to evaluate the effects of different defined culture media (containing 100 U/ml penicillin and 100 µg/ml streptomycin and 5% FBS) on parasite vitality and viability. These effects were visually determined as phenotypic alterations in parasite motility, shape and translucence [76]. Time-lapse image recordings, taken at day 7 of the incubation, are provided as Videos S1, S2, S3, S4, S5, S6, S7 and provide the best visual medium to compare and contrast the worm responses to the incubation conditions. Still images from these experiments are also presented in Figure 1. By the seventh day of incubation, relative to Basch medium 169, DMEM (high glucose), F-12, Liebowitz and M199 media had generated varying degrees of deformed, rounded and/or darkened parasites, often accompanied by slowed and less dynamic motility. In RPMI and Schneider's media, additional and more severe effects (degenerating parasites and widespread death) were noted, which appeared as early as day 2 of the incubation and progressed to approximately >80% parasite mortality by day 7. For RPMI, this was also case when the medium was exchanged every 2 days. In contrast, in Basch medium 169, parasites were more uniform in translucency, shape and motility with less than 10% mortality at day 7. Based on these results, therefore, we employed Basch medium 169 for all further experiments.

### The issue of carry-over contaminating DNA in accurately measuring RNAi

In spite of the mandatory DNase treatment used to remove PCR-derived DNA from dsRNA preparations, the possibility existed that residual DNA would nevertheless serve as template for qPCR should the qPCR primers be designed to amplify within the sequence used to synthesize dsRNA [55], [74]. This would result in an apparent lack of, or less efficient, transcript knockdown. That this is a legitimate concern (and even with best practice to purify dsRNA according to the appropriate protocol) is shown in Figure S1 using ‘purified’ dsRNA to the tegument-associated SmCB2 transcript as an example. A concentration-dependent amplification resulted (Figure S1A) when qPCR primers (Primer set B) were positioned within the sequence employed as template for reverse transcription (Figure S1B). In contrast, primer set A, that targeted an area 5′ of the dsRNA template sequence, produced no such amplification.

### Direct toxicity of dsRNA to schistosomula, and dose- and time-dependency of RNAi

**Figure 2:**
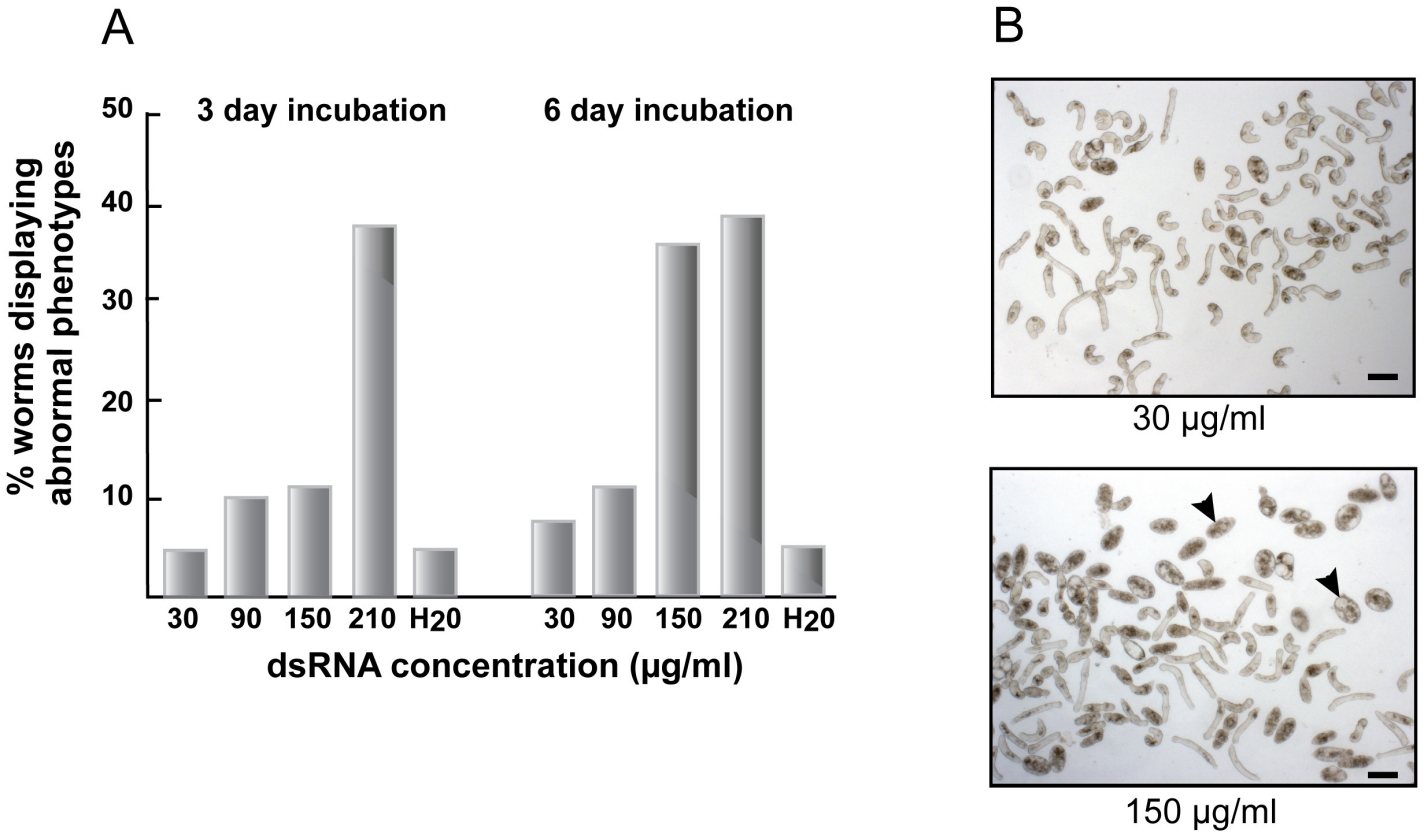
Direct toxicity by dsRNA to newly transformed schistosomula. Parasites were incubated in a total volume of 1 ml Basch Complete Medium with various concentrations of dsRNA (aliquots of 70 µl water) to *Discosoma* sp. mCherry [84]. At days 3 and 6, three photographic fields of view per dsRNA concentration were taken and those parasites displaying an obvious loss of shape and morphological integrity counted. The data shown in (A) are the averages of those counts expressed as a percentage of the total number of worms. SD values were less than 10% of the mean. (B) Images of parasites exposed to different concentrations of dsRNA by day 6 of the incubation: arrowheads indicate examples of distressed worms induced by 150 µg/ml dsRNA. Data are representative of two experiments. Similar results were observed with dsRNA to SmCB1. Scale bars represent 100 µm.

**Figure 3:**
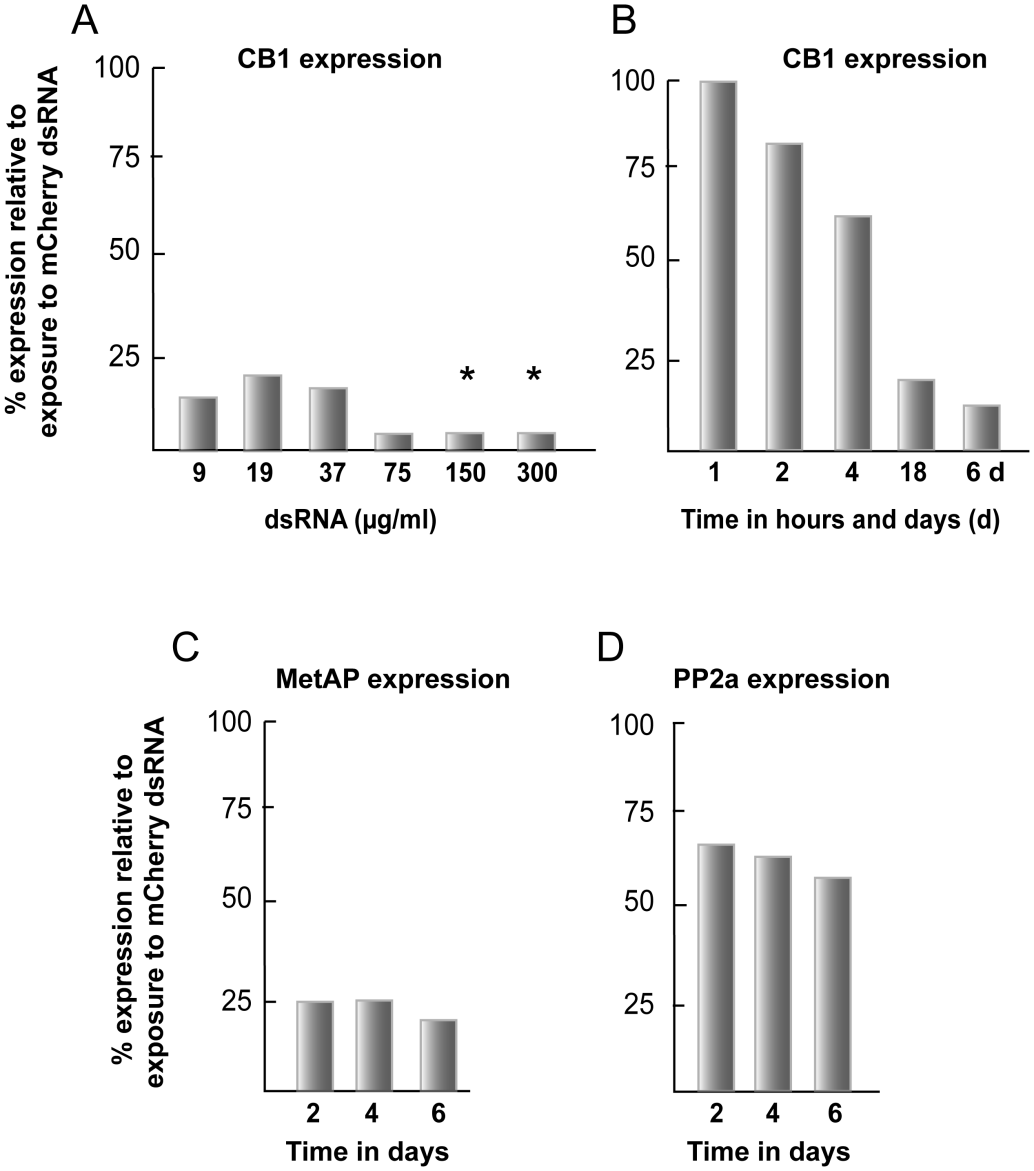
Dose and time dependency of RNAi in newly transformed schistosomula. Parasites were incubated with (A) varying concentrations of dsRNA or (B, C and D) 30 µg/ml dsRNA for various periods up to 6 days. RNAi was measured by qRT-PCR and data expressed relative to those following parasite exposure to schistosome-unspecific mCherry dsRNA. Asterisks in (A) indicate direct dsRNA toxicity to the parasites (see text and Figure 2 for details). Each sample was tested in duplicate and representative data from two experiments are shown.

Direct toxicity to schistosomula by dsRNA was evident as greater proportions of parasites that displayed abnormal phenotypes [76] such as rounding, darkening, slowed motility and outright death, or a combination thereof (Figure 2). Using dsRNA to mCherry (or CB1, not shown) and at the highest concentration tested of 210 µg/ml, more than 35% of the parasites appeared abnormal by days 3 and 6 of the incubation (Figure 2A). At a concentration of 150 µg/ml, a marked increase in the percentage of distressed parasites was measured between days 3 (12%) and 6 (35%; Figure 2A and 2B, lower panel). Concentrations below this (30 and 90 µg/ml) did not increase the percentage of abnormal parasites above the 12% background for the duration of the experiment. Thus, we interpret the data to suggest that the upper concentration limit for dsRNA, without incurring obvious parasite distress, is approximately 90 µg/ml. Operationally, we chose 30 µg/ml dsRNA to provide an increased margin of safety and based on the dsRNA dose-dependency tests for CB1 (Figure 3A), MetAP, NEC and PP2a (not shown) indicating that 30 µg/ml was sufficient for maximal RNAi. We chose a period of 6 days for co-incubating dsRNA and parasites as it was sufficient to register maximal transcriptional suppression of CB1, MetAP and PP2a (Figure 3B, C and D, respectively).

### RNAi, including more than one transcript target, is selective, however, its efficiency is gene-dependent

**Figure 4:**
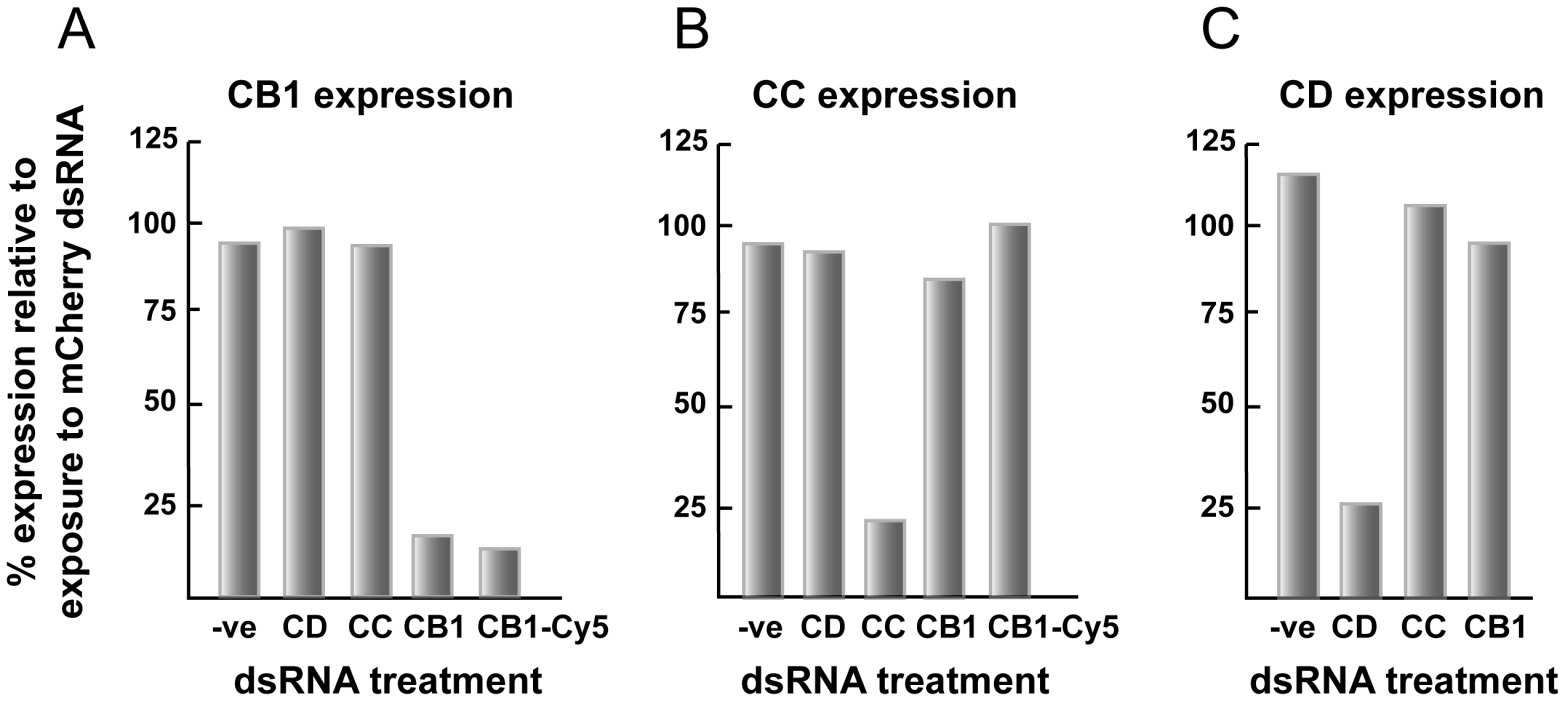
RNAi of gut-associated gene transcripts in newly transformed schistosomula is robust and selective. Parasites were incubated for 6 days with 30 µg/ml of each of the indicated dsRNA preparations. RNAi was measured by qRT-PCR and data expressed relative to those following parasite exposure to schistosome-unspecific mCherry dsRNA. A negative control (−ve) in which parasites had not been exposed to dsRNA was also included. For each of the targeted genes, (A) SmCB1, (B) SmCC and (C) SmCD, the RNAi effect was selective among the pool of gut-associated transcripts examined. Selective RNAi of SmCB1 was also achieved with 30 µg/ml Cy5-linked dsRNA. Each sample was tested in duplicate and representative data from two experiments are shown.

Transcripts for the gut-associated proteins CB1, CC and CD are sensitive to RNAi and are robustly suppressed by >75% (Figure 4A, B and C, respectively). Importantly, the effect is selective for the intended mRNA within the pool of gut transcripts examined. Attachment of the fluorescent Cy5 label to CB1-(Figure 4A) or CC-(not shown) specific dsRNA does not interfere with suppression, or the degree of suppression, of the cognate mRNA. With non-labeled dsRNA targeting CB1, CC and CD, the RNAi effect was long-term, remaining constant out to 3 weeks of incubation without exchanging either culture medium or dsRNA (not shown). For CB1, long-term suppression (>30 days of incubation) of transcript has been previously noted for schistosomula exposed at 3 h [53] and 7 days post-transformation [96].

**Figure 5:**
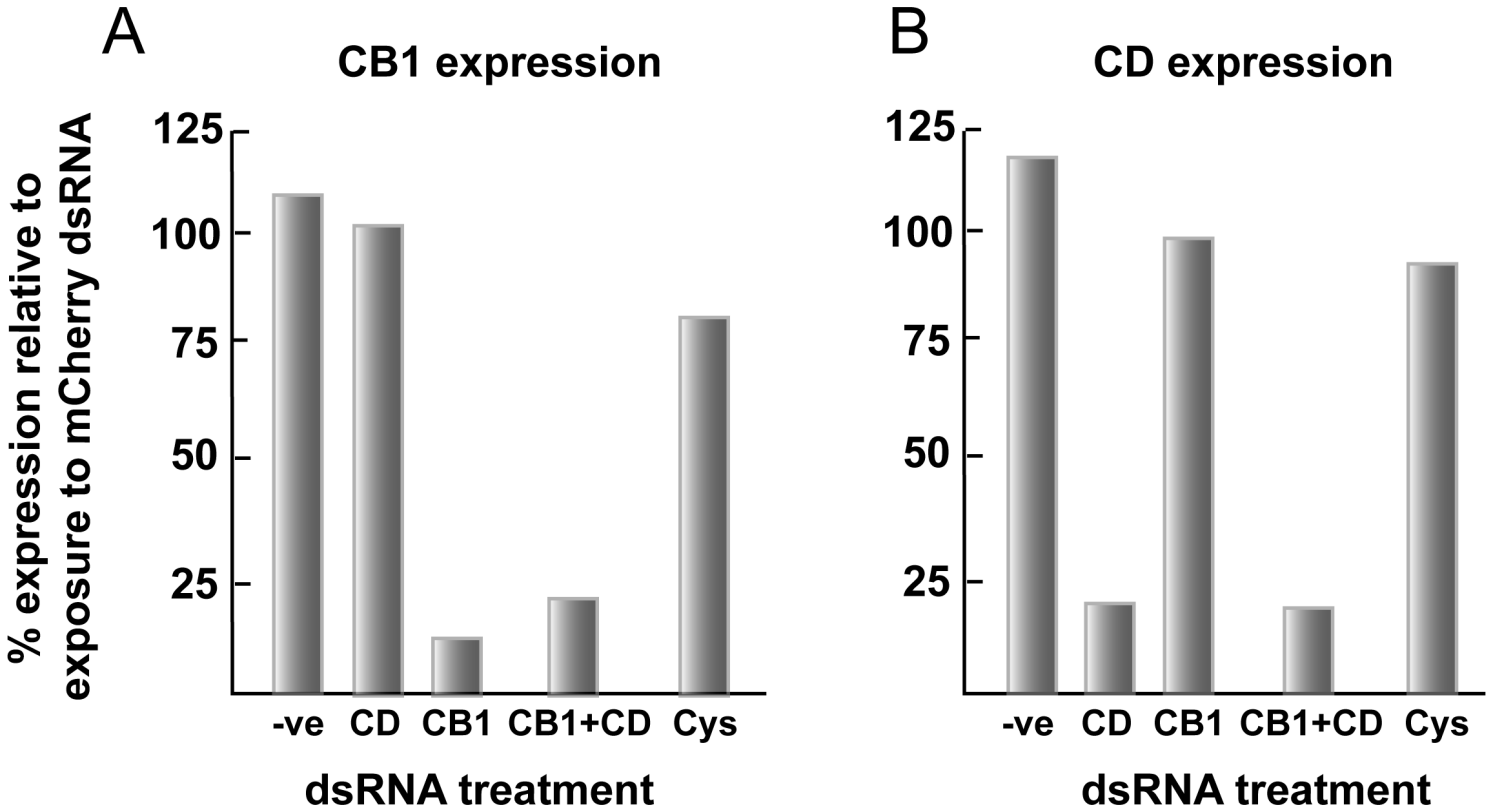
RNAi of more than one gut-associated gene transcript is robust and selective. Newly transformed schistosomula were incubated for 6 days with either 30 or 60 µg/ml of the indicated dsRNA preparations depending on whether single or double transcript suppression of SmCB1 (A) or SmCD (B) was being attempted. RNAi was measured by qRT-PCR and data expressed relative to those following parasite exposure to schistosome-unspecific mCherry dsRNA at 60 µg/ml. Included as controls were parasites that had not been exposed to dsRNA (−ve) or that had been co-incubated with dsRNA to the cysteine protease inhibitor cystatin (Cys). Each sample was tested in duplicate and representative data from two experiments are shown.

As exemplified by CB1 and CD (Figure 5A and B, respectively), RNAi of more than one transcript at the same time is possible in schistosomula (30 µg/ml dsRNA per transcript target), in this case with >75% suppression. As found for RNAi of single transcripts, the effect is again selective within the pool of transcripts examined. For these experiments, the transcript for cystatin (Cys), a cysteine protease inhibitor (AY334553.1) [97], was included as a non-targeted transcript control.

**Figure 6:**
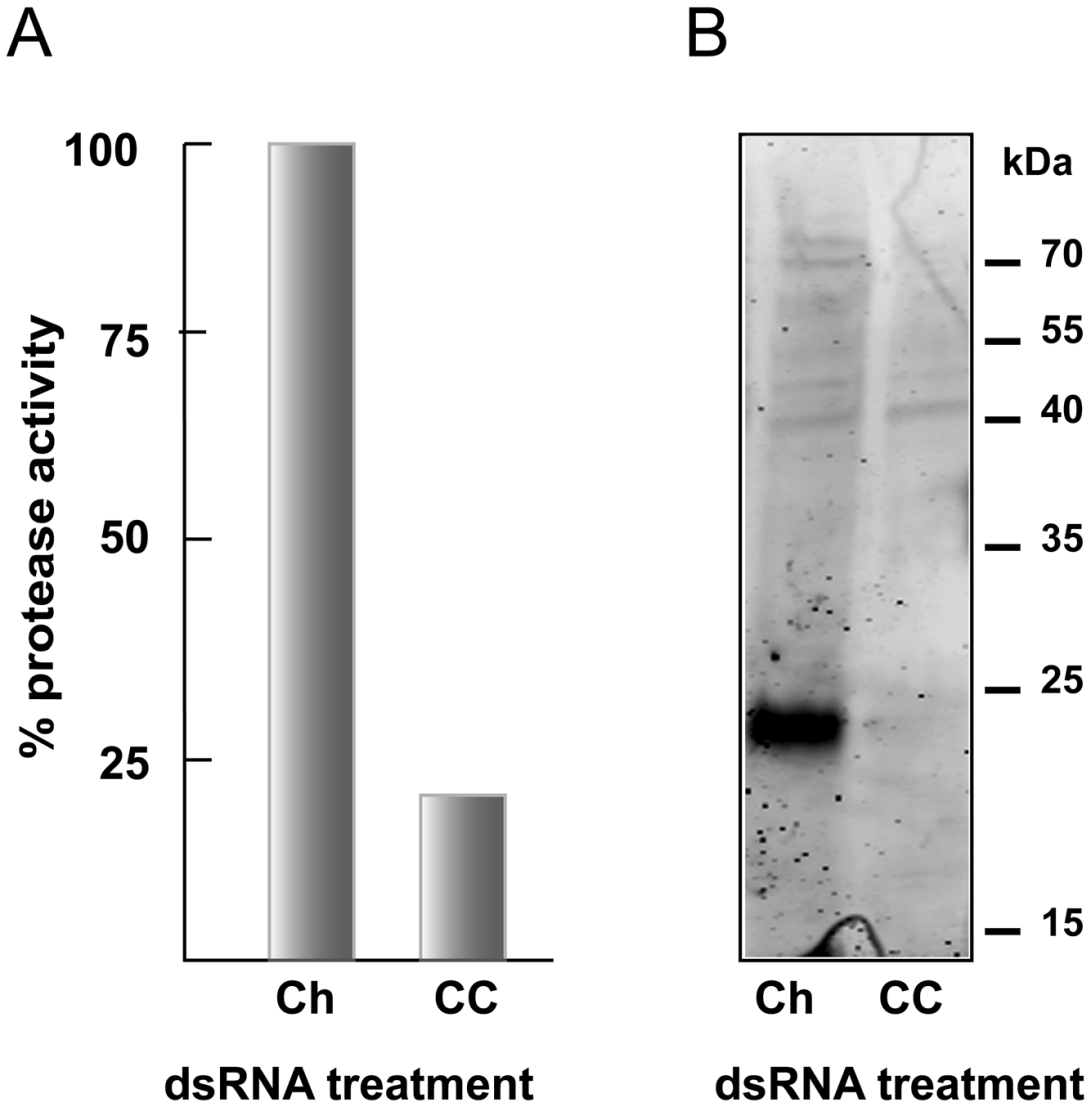
RNAi of SmCC is accompanied by decreased translation product measured as proteolytic activity. (A) Activity in extracts of schistosomula that had been co-incubated for 6 days with 30 µg/ml mCherry (Ch) or SmCC (CC) dsRNA was measured with the cathepsin C-selective peptidyl substrate, GR-AMC. Worm extracts were normalized to 0.4 µg of protein per reaction and the proteolytic activity in extracts from parasites exposed to mCherry dsRNA was set to 100%. (B) Reactivity with the fluorescent cathepsin C-selective activity-based probe (bodipy-labeled FY01; [91]) as assessed by SDS-PAGE. Worm extracts were normalized to 2 µg per labeling reaction. The fluorescent signal at 24 kDa is lost subsequent to exposure to CC-specific dsRNA. Data in (A) are the average of triplicate values (SD values were less the 5% of the mean). Representative data from two experiments are shown.

Confirmation of RNAi at the protein level was sought for CC (Figure 6). A 6-day co-incubation of schistosomula and dsRNA targeting CC realized a knockdown of the translation product, measured either as an 80% decrease in the peptidolytic cleavage of the cathepsin C-selective substrate, GR-AMC (Figure 6A) or as a loss of the proteolytic signal (at approximately 24 kDa) using the specific active site-directed probe, bodipy-labeled FY01 (Figure 6B). Activity in the presence of GR-AMC was not inhibited by the general cysteine protease inhibitor, K11777, or by CA-074 and Z-Phe-Phe-DMK that target cathepsins B and L, respectively. This indicates that only cathepsin C activity was being measured. For CB1, a similar absence of protein was recorded after dsRNA application using immunoblotting with monospecific antibodies (not shown).

**Figure 7:**
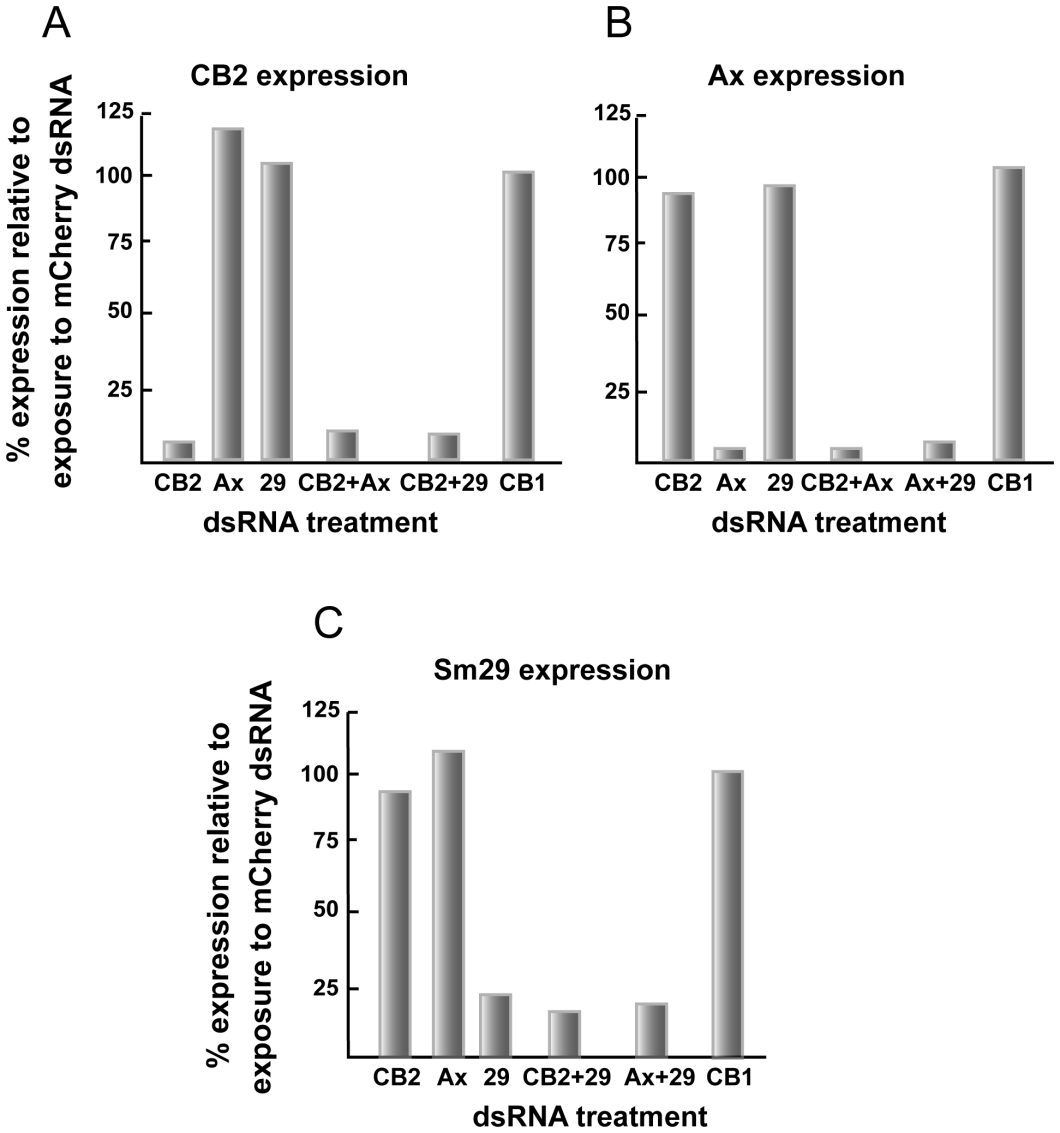
RNAi of one, or more than one, tegument-associated gene transcript is robust and selective. Newly transformed schistosomula were incubated for 6 days with either 30 or 60 µg/ml of the indicated dsRNA depending on whether single or double transcript suppression was being attempted. RNAi was measured by qRT-PCR and data expressed relative to those following parasite exposure to schistosome-unspecific mCherry dsRNA at 60 µg/ml. For each of the targeted genes, (A) SmCB2, (B) SmAx (annexin) and (C) Sm29, the RNAi effect was selective among the pool of tegument transcripts examined and also with respect to the transcript for the gut-associated protease, SmCB1, used as a ‘tissue control’. Each sample was tested in duplicate and representative data from two experiments are shown.

Robust and selective single and double knockdowns (>75%) were also possible for the tegument transcript targets CB2, annexin and Sm29 using 30 µg/ml dsRNA per transcript (Figure 7A, B and C, respectively). This was the case within the pool of tegument transcripts examined and as compared to the gut-associated CB1 employed as a ‘tissue control’.

**Figure 8:**
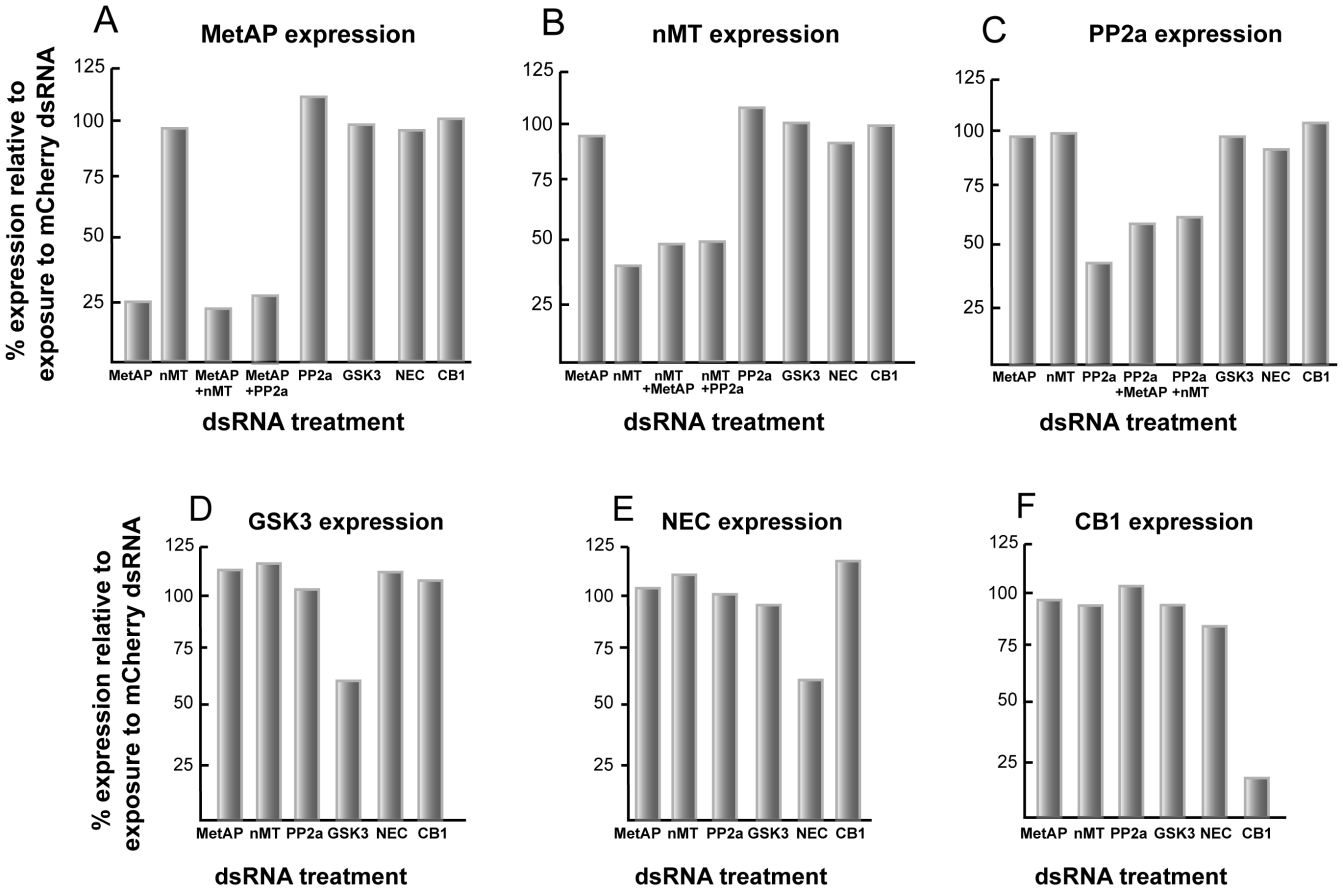
For 5 other gene transcripts, RNAi of one, or more than one target is selective, but efficiency varies. Newly transformed schistosomula were incubated for 6 days with either 30 or 60 µg/ml of the indicated dsRNA depending on whether single or double transcript suppression was being attempted. RNAi was measured by qRT-PCR and data expressed relative to those following parasite exposure to schistosome-unspecific mCherry dsRNA at 60 µg/ml. For each of the targeted genes, (A) SmMetAP, (B) SmnMT (C) SmPP2a, (D) SmGSK3 and (E) SmNEC, the RNAi effect was selective among the pool of genes examined and also with respect to the transcript for the gut-associated protease, SmCB1, used as a ‘tissue control’. Likewise, the corollary experiment (F) indicates that RNAi of CB1 does not affect transcript levels of the other gene targets. Each sample was tested in duplicate and representative data from two experiments are shown.

For the 5 remaining genes that are considered to be more generally distributed (MetAP, nMT, PP2a, GSK3, and NEC), selective single and double (for MetAP, nMT and PP2a) knockdowns were generated with 30 µg/ml dsRNA per transcript (Figure 8). However, compared to the gut and tegument targets, the efficiency of knockdown was more variable. Thus, a decrease of approximately 75% was recorded for MetAP (Figure 8A); whereas for nMT and PP2a, decreases of between 40 and 60% were obtained (Figure 8B and C). GSK3 and NEC transcripts were more resistant to RNAi with maximum decreases of approximately 40% (Figure 8D and E). RNAi of each target did not affect levels of CB1 transcript used as a ‘tissue control’. Likewise, RNAi of CB1 did not affect transcript levels of the other gene targets (Figure 8F). Doubling or tripling the concentration to 90 µg/ml dsRNA did not improve RNAi (not shown), nor was it the case that transcript suppression was greater earlier in the 6-day incubation period (as shown when processing samples for transcript levels of MetAP and PP2a at days 2 and 4 of the incubation; Figure 3C). Thus, in the present experimental system, the RNAi recorded seems to be the maximal achievable.

No striking or obvious phenotypes were observed upon RNAi of any of the 11 gene transcripts over the 6-day incubation period. For the 3 gut-associated and 5 *in silico*-predicted essential genes, this was also the case after 3 week co-incubations and whether or not the culture medium and dsRNA were exchanged weekly (not shown). For CB1 and CD, the same was also true in the presence of washed erythrocytes and was independent of whether single or double RNAi was employed (not shown).

### The contribution by electroporation to RNAi is minor and may depend on the tissue localization of the target

Electroporation has been employed as part of a protocol to deliver nucleic acids to both adult schistosomes and schistosomula e.g., [53], [57], [96], [98]. Our experience with electroporation has entailed some risk of contamination of parasite culture consequent on the extra handling required to perform this technique. These issues are of logistical importance when streamlining larger scale RNAi experiments. We, therefore, devised a series of experimental conditions for selected gene targets to determine the contribution to RNAi by electroporation as compared to simple co-incubation.

**Figure 9:**
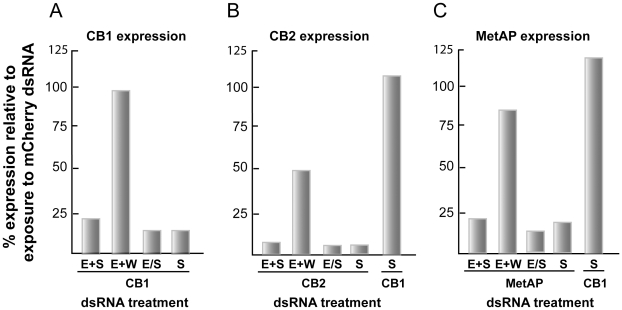
Electroporation is not necessary for maximal RNAi in newly transformed schistosomula. Parasites were exposed to 30 µg/ml dsRNA for representative gut (SmCB1), tegument (SmCB2) and otherwise (SmMetAP) targets and then incubated for 6 days. Experimental parameters were as follows: E+S, electroporation with dsRNA followed by soaking (i.e., co-incubation with dsRNA); E+W, electroporation with dsRNA then washing worms 10 times to remove extraneous dsRNA and followed by soaking; E/S, electroporation without dsRNA and then soaking in the presence of dsRNA; S, soaking with dsRNA without prior electroporation. RNAi was measured by qRT-PCR and data expressed relative to those following parasite exposure to schistosome-unspecific mCherry dsRNA. Simple soaking of dsRNA targeting either SmCB1 (A) or SmMetAP (C) was sufficient to achieve maximal RNAi; electroporation had a minor effect. In contrast, for the tegumental protease, SmCB2, (B) (also for SmAx and Sm29, not shown), electroporation mediated a greater effect, but still not as pronounced as that produced by simple co-incubation. In both (B) and (C), levels of SmCB1 transcript, employed as a ‘tissue control’, were unaffected. Each sample was tested in duplicate and representative data from two experiments are shown.

Co-incubation of dsRNA with schistosomula or electroporation in the presence or absence of dsRNA followed by co-incubation of dsRNA with parasites led to robust transcript suppression (>75%) for each the tissue-representative targets examined, namely CB1 (gut), CB2 (tegument) and MetAP (otherwise; Figure 9). In contrast, when worms that had been electroporated in the presence of dsRNA were then immediately washed 10 times prior to incubation in the absence of dsRNA, the RNAi effect was more limited. Thus, for the gut proteases CB1 (Figure 9A), CC and CD (not shown), transcript levels were decreased by between 0 and 15% relative to mCherry. Similarly, only an approximate 20% decrease in message was recorded for MetAP when the contribution of electroporation to RNAi was isolated (Figure 9C). However, for the tegument targets, CB2 (Figure 9B), annexin and Sm29 (not shown), electroporation alone generated a more apparent RNAi of approximately 50%. This suggests that tegument targets may be more amenable to electroporation, perhaps facilitated by their greater proximity to the transducing electrical field generated by electroporation. Yet, overall, the contribution of electroporation, when isolated from the other experimental parameters, to the RNAi effect is relatively minor, and consequently could be omitted as an operational parameter when streamlining larger-scale screens with dsRNA. We note, however, that what may apply for optimal delivery of long dsRNA may not be the case for siRNA.

### The gut as a mediator of RNAi in newly transformed schistosomula

**Figure 10:**
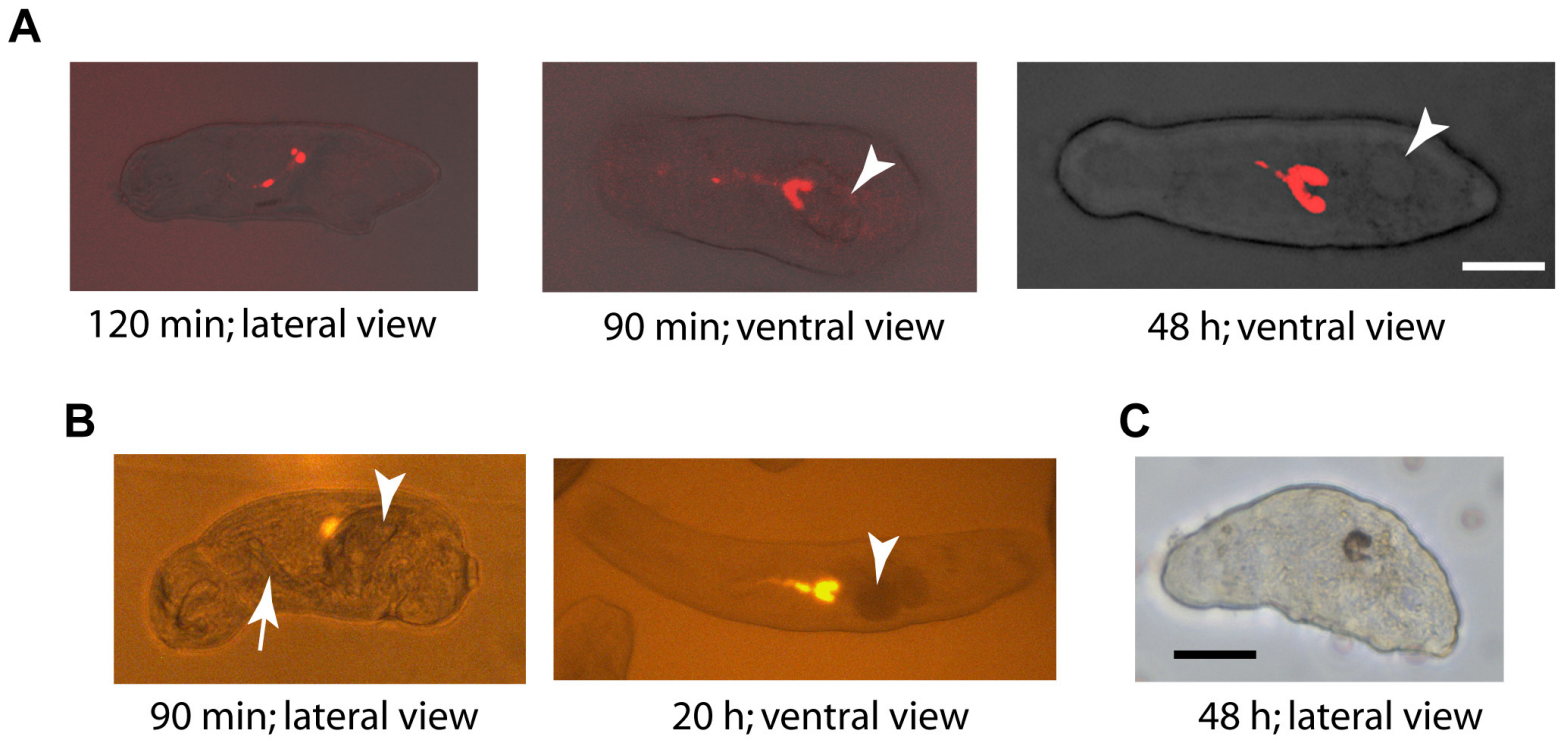
The gut of newly transformed schistosomula is active and likely facilitates RNAi. After their preparation from cercariae, schistosomula were incubated with (A) 30 µg/ml Cy5-linked dsRNA targeting SmCB1, (B) 200 µg/ml recombinant mCherry protein or (C) 5 µl washed and packed human erythrocytes. For each image, the anterior end of the parasite is leftmost and times stated are post-preparation from cercariae. Both the Cy5-linked dsRNA and mCherry emit a strong fluorescent signal that quickly accumulates in the gut including in the two terminal cecal chambers. The arrow and arrowheads indicate the acetabular ‘gland ducts’ and acetabular ‘glands’, both being distinct from the developing gut. In (C), brown material, derived from the digestion of hemoglobin, highlights the parasite gut. Images were captured using: (A) a Zeiss LSM 510 META confocal microscope and a 633 nm laser for excitation; (B) a Zeiss Axiovert 40 CFL inverted microscope using the Filter Set 20 (ex-546; em-575-640) and connected to a Zeiss AxioCam HRc digital camera and (C) a Zeiss Axiovert 40 C inverted microscope connected to a Zeiss AxioCam MRc. Both AxioCAms were operated by AxioVision 40, version 4.5.0.0, software. Scale bars represent 20 µm.

Given the above data suggesting that simple co-incubation of newly transformed parasites and dsRNA is sufficient to mediate RNAi, we then asked the question whether the gut serves as a route of entry for dsRNA into the schistosome. The question is relevant in view of previous discussions to the effect that the parasite mouth is not open until about day 7 of the incubation (i.e., indicative of a non-functioning gut) and, therefore, cannot facilitate RNAi in younger schistosomula [96]. Using Cy5-labeled dsRNA to CB1, CC or mCherry, we demonstrate that the gut of schistosomula takes up and concentrates exogenous material within minutes of their mechanical preparation (Figure 10; results with CB1 dsRNA shown). Accumulation of the dye was evident along the gut and in the two terminal cecal chambers by 90 min post-transformation (Figure 10A). At the same time-point, a fluorescent signal from accumulated mCherry protein could be detected (Figure 10B). For both probes, the signal remained visible during the incubation period of 6 days illuminating the gut ceca as they elongated posteriorly over time. Consistent with this rapid onset of gut activity, we could already measure RNAi of CB1 at between 2 and 4 h post-incubation with dsRNA, an effect that increased as a function of time (Figure 3B). Finally, we could detect hemoglobin-derived pigment in the gut of 2 day-old schistosomula co-incubated with erythrocytes (Figure 10C). Taken together, the results suggest that the schistosomulum gut is functionally active within minutes of their preparation from cercariae and seems a likely route through which RNAi is mediated.

## Discussion

Large-scale RNA-dependent gene silencing is an informative reverse genetic platform to interrogate decreased or loss of gene function at the protein, pathway, cell and organism levels. RNAi can also aid the identification of drug targets for the treatment of a variety of disease states [99], [100], [101], including those caused by pathogens [102], [103]. For schistosomes, a number of key components (comprehensive genome and transcriptome data, interrogable databases, and both *in silico* and experimental evidence of the RNAi phenomenon) are now in place to facilitate the implementation of larger scale RNAi-based screening campaigns.

In advance, we investigated the sensitivity and selectivity of RNAi for a set of 11 genes, which contained representatives from different tissue types – gut, tegument and otherwise. We employed long (approximately 500 bp) dsRNA rather than siRNA as the former can be produced at scales sufficient for performing multiple experiments and based on the assumption that the variety of siRNAs generated from the parent dsRNA would provide for as much silencing potential as possible. We focused on mechanically transformed schistosomula that are both relevant to parasitism in humans and, compared to adult parasites, available in greater numbers for automated high throughput screening [76]. In addition, issues of operational efficiency and simplicity were also considered, including, choice of culture medium, transfection strategy, dsRNA time- and dose-dependency and dosing limits. It was during these studies that the gut was identified as a likely route for entry of dsRNA into the newly transformed parasite.

The question of which culture medium to use for optimal maintenance of schistosomula has not been directly evaluated since the pioneering culture work of Cheever and Weller, and Clegg in the 1950s and 60s [104], [105], and Basch in the early 1980's [78]. The issue is also relevant to chemical drug discovery programs that involve co-incubation of schistosomula with compounds and the recording of phenotypes [76]. Of the seven media tested here (Basch Medium 169, DMEM, F-12, Liebowitz's, M199, RPMI 1640 and Schneider's), all except Basch Medium 169 induced abnormal phenotypes amounting to darkened and/or distorted parasites often accompanied by slowed and less dynamic motility. In the case of Schneider's and RPMI 1640 media, widespread death occurred. Of note was the poor survival of schistosomula in RPMI 1640, a medium that has been previously employed for a number of RNAi studies with schistosomula [52], [65], [96], [98]. Our concern was that the high background mortality observed here would make determinations of an RNAi effect, either at the mRNA level or, certainly, at the phenotypic level, difficult. Yet, despite the poor survival in RPMI, RNAi of CB1 transcript could be decreased to levels similar to those achieved in Basch Medium 169 (data not shown) - a finding consistent with the effect observed previously. Nevertheless, based on the present media tests, we employ Basch Medium 169 as the medium of choice for maintenance of *S. mansoni* schistosomula as mortality remained at approximately 10% out to 4 weeks of incubation. Indeed, consistent with the earlier report [78], we have been able to maintain parasites long enough (about 8 months) in Basch Complete Medium 169 to the point of pairing of males and females (unpublished data).

Key concerns in any large scale screening campaign are the occurrence and extent of off-targeting. The possibility of such phenomena operating in schistosomes has been raised in a recent report involving RNAi of 32 genes expressed in *S. mansoni* mother sporocysts [74], [75]. For the smaller set of 11 genes studied here in schistosomula, we did not observe off-targeting which is encouraging for larger scale studies. RNAi, including suppression of more than one transcript, was selective to the intended target(s) among and between the different pools of gut, tegument and generally distributed transcripts investigated. The finding is also important as it expands upon previous data for smaller numbers (1 to 3) of genes of interest [55], [65] that demonstrated RNAi specificity relative to schistosome-unspecific dsRNA preparations used as controls. The apparent lack of off-targeting in newly transformed schistosomula is consistent with data from similarly designed experiments for a pool of gut-associated proteases in 3-week old *S. mansoni* [54]. That the contemporaneous and selective suppression of more than one gene is possible in schistosomula could prove useful in interrogating functional redundancy within protein systems. An example of such is the network of gut proteases that digest host proteins [54], [79]. Multiple RNAi of component proteases may help reveal the extent of redundancy and highlight cooperative relationships in how host proteins are processed.

For both the gut and tegument genes studied, knockdown efficiency, including after double-targeting, was at least 75%. For the gut proteases, CC and CB1, transcript knockdown was mirrored by a substantial decrease or loss of translation product as determined biochemically with a specific substrate and active site-directed probe for CC, and a monospecific anti-serum to CB1. The robust suppression of CB1 and CD recorded here is consistent with previous studies using schistosomula [53], [55], [96] and 3 week-old sub-adult worms [54]. Though our observations did not include measurements of worm size to statistically discriminate dsRNA-induced phenotypic alterations [53], [55], we noted no striking abnormalities in parasites co-incubated for 3 weeks with dsRNA to CB1 and/or CD in the presence or absence of erythrocytes. This included a previous observation of red-colored, rather than the normally black, gut contents in a population of schistosomula exposed to CD-dsRNA in the presence of erythrocytes– a finding that prompted the suggestion that diminished CD activity was responsible for the phenotype [55]. At this time, we cannot explain the difference in phenotypic outcomes, however, we observe that the color of gut contents of individual schistosomula normally varies from black to brown to red when cultured in the presence of erythrocytes.

The robust RNAi of gut and tegument targets contrasts with the more variable efficiencies recorded for the 5 gene targets considered to be more generally distributed in the parasite and that had been predicted *in silico* to be essential [46]. Thus, the efficiency of RNAi ranged from approximately 75% (MetAP) to approximately 40% for GSK3 and NEC. We speculate that the localization of gene expression is in part responsible for this variation whereby genes expressed in the gut and tegument are more likely to be efficiently suppressed given their proximity to the external environment containing dsRNA.

Such gene-to-gene variability in sensitivity to RNAi is likely inherent when, under a standardized set of conditions such as those under investigation here, not all gene transcripts will respond to the same degree. Variable gene responses to RNAi have also been recently described for *S. mansoni* mother sporocysts [74], [75] and schistosomula [65] exposed to dsRNA. Transcripts not decreased below certain critical thresholds will contribute to the lack of obvious phenotypes arising. This could also explain why targeting the 5 genes that had been predicted *in silico* as being essential to *S. mansoni* [46] produced no gross phenotypic alterations, even at near toxic dsRNA concentrations or over an extended 3-week co-incubation period. Of course, this is aside from the more direct possibility that the genes are not, after all, essential to survival of the parasite or the particular developmental stage in question. In addition, and as discussed previously [51], even for genes that are sensitive to RNAi, issues such as developmental plasticity of the schistosome and functional redundancy in protein function, pathways or networks will limit the number of readily observable phenotypes. Yet, given these considerations, literature reports of striking phenotypes post-RNAi in schistosomula (e.g., [59], [63]) would encourage continued loss-of-function investigations, especially in view of the imperative to identify new drug targets.

The finding that co-incubation of newly transformed parasites and dsRNA, without the need for electroporation (i.e., trans-tegumental delivery of dsRNA), is sufficient to induce robust RNAi focused our attention on the parasite gut in dsRNA uptake. By 90 min post-production from cercariae we could observe a concentration of fluorescent macromolecules above background in the gut that was indicative of rapid and active ingestion of exogenous material by the parasite. This gut activity during transformation from the cercarial state is consistent with electron microscopy studies demonstrating rearrangements of the esophageal environment [106] and earlier observations that transforming parasites engulf culture fluid [107]. In parallel with the ingestion of fluorescent materials, we could measure decreased CB1 transcript 2 to 4 hours after co-incubation of worms with specific dsRNA suggesting that dsRNA, upon ingestion, is both rapidly absorbed and processed to initiate mRNA suppression. It is possible that the tegument or other organ systems such as the acetabular glands [96] or nephridiopore [108] also act as entry points for dsRNA in nascent schistosomula, however, the present results suggest that the gut should also be considered as a key contributor. Finally, the observation of blood products in 2 day-old schistosomula reinforces the evidence that the gut is quickly put to work by the parasite in the acquisition of nutrients.

The data reported herein that newly transformed schistosomula are amenable to RNAi is promising in view of previous reports observing less efficient or inconsistent suppression (of CB1) in parasites <7days old [53], [96]. The underlying reasons for these apparent differences in RNAi kinetics are unclear and could be reviewed as part of a broader discussion on assay development and refinement. From the perspective of screen design, however, it is logistically much simpler to move directly from ‘snails to screens’ employing newly transformed schistosomula without factoring for both the extra time and handling required for older parasites, particularly as they seem to be more susceptible to handling damage than newly transformed schistosomula (unpublished data).

## Supporting Information

Figure S1 The influence of qPCR primer positioning on the amplification of residual PCR product carried over from dsRNA synthesis. There is no amplification of residual PCR product (Panel A) when primer pairs (Primer set A) are positioned upstream from that part of the open reading frame of SmCB2 used to synthesize dsRNA (Panel B). In contrast, Primer set B (positioned within the sequence used to generate dsRNA) amplifies residual PCR product in a concentration-dependent manner that would negatively impact the apparent efficiency of RNAi as measured by qRT-PCR. Each sample was tested in duplicate and representative data from two experiments are shown.
(0.35 MB TIF)

Table S1 List of primer sequences employed for dsRNA synthesis (the T7 DNA polymerase binding motif is shown in bold characters) and quantitative real-time (qRT)-PCR.
(0.03 MB XLS)

Video S1 Phenotypic responses of *S. mansoni* schistosomula upon incubation in Basch Medium 169. Parasites were maintained for 7 days in medium containing 5% FBS, and 100 U/ml penicillin and 100 µg/ml streptomycin. Time-lapse recordings were captured with a Zeiss Axiovert 40 C inverted microscope (10× magnification) connected to a Zeiss AxioCam MRc digital camera that was under the control of AxioVision 40 version 4.5.0.0 software. Note that parasites in this medium appear most uniform in translucency, shape and motion with the least background mortality.
(3.66 MB MOV)

Video S2 Phenotypic responses of *S. mansoni* schistosomula upon incubation in DMEM (high glucose) medium. Parasites were maintained for 7 days in medium containing 5% FBS, and 100 U/ml penicillin and 100 µg/ml streptomycin. Time-lapse recordings were captured with a Zeiss Axiovert 40 C inverted microscope (10× magnification) connected to a Zeiss AxioCam MRc digital camera that was under the control of AxioVision 40 version 4.5.0.0 software.
(3.53 MB MOV)

Video S3 Phenotypic responses of *S. mansoni* schistosomula upon incubation in F-12 medium. Parasites were maintained for 7 days in medium containing 5% FBS, and 100 U/ml penicillin and 100 µg/ml streptomycin. Time-lapse recordings were captured with a Zeiss Axiovert 40 C inverted microscope (10× magnification) connected to a Zeiss AxioCam MRc digital camera that was under the control of AxioVision 40 version 4.5.0.0 software.
(3.47 MB MOV)

Video S4 Phenotypic responses of *S. mansoni* schistosomula upon incubation in Liebowitz's medium. Parasites were maintained for 7 days in medium containing 5% FBS, and 100 U/ml penicillin and 100 µg/ml streptomycin. Time-lapse recordings were captured with a Zeiss Axiovert 40 C inverted microscope (10× magnification) connected to a Zeiss AxioCam MRc digital camera that was under the control of AxioVision 40 version 4.5.0.0 software.
(3.44 MB MOV)

Video S5 Phenotypic responses of *S. mansoni* schistosomula upon incubation in M199 medium with Earle's Salts. Parasites were maintained for 7 days in medium containing 5% FBS, and 100 U/ml penicillin and 100 µg/ml streptomycin. Time-lapse recordings were captured with a Zeiss Axiovert 40 C inverted microscope (10× magnification) connected to a Zeiss AxioCam MRc digital camera that was under the control of AxioVision 40 version 4.5.0.0 software.
(4.30 MB MOV)

Video S6 Phenotypic responses of *S. mansoni* schistosomula upon incubation in RPMI 1640 medium. Parasites were maintained for 7 days in medium containing 10 mM HEPES, 2 mM glutamine, 5% FBS, and 100 U/ml penicillin and 100 µg/ml streptomycin. Time-lapse recordings were captured with a Zeiss Axiovert 40 C inverted microscope (10× magnification) connected to a Zeiss AxioCam MRc digital camera that was under the control of AxioVision 40 version 4.5.0.0 software.
(2.81 MB MOV)

Video S7 Phenotypic responses of *S. mansoni* schistosomula upon incubation in Schneider's medium. Parasites were maintained for 7 days in medium containing 5% FBS, and 100 U/ml penicillin and 100 µg/ml streptomycin. Time-lapse recordings were captured with a Zeiss Axiovert 40 C inverted microscope (10× magnification) connected to a Zeiss AxioCam MRc digital camera that was under the control of AxioVision 40 version 4.5.0.0 software.
(2.63 MB MOV)
