# Supplementary figures and images for: RNA Interference in Schistosoma mansoni Schistosomula: Selectivity, Sensitivity and Operation for Larger-Scale Screening
Source: PLoS Negl Trop Dis. 2010 Oct 19;4(10):e850. doi: 10.1371/journal.pntd.0000850 (PMC2957409; doi:10.1371/journal.pntd.0000850)

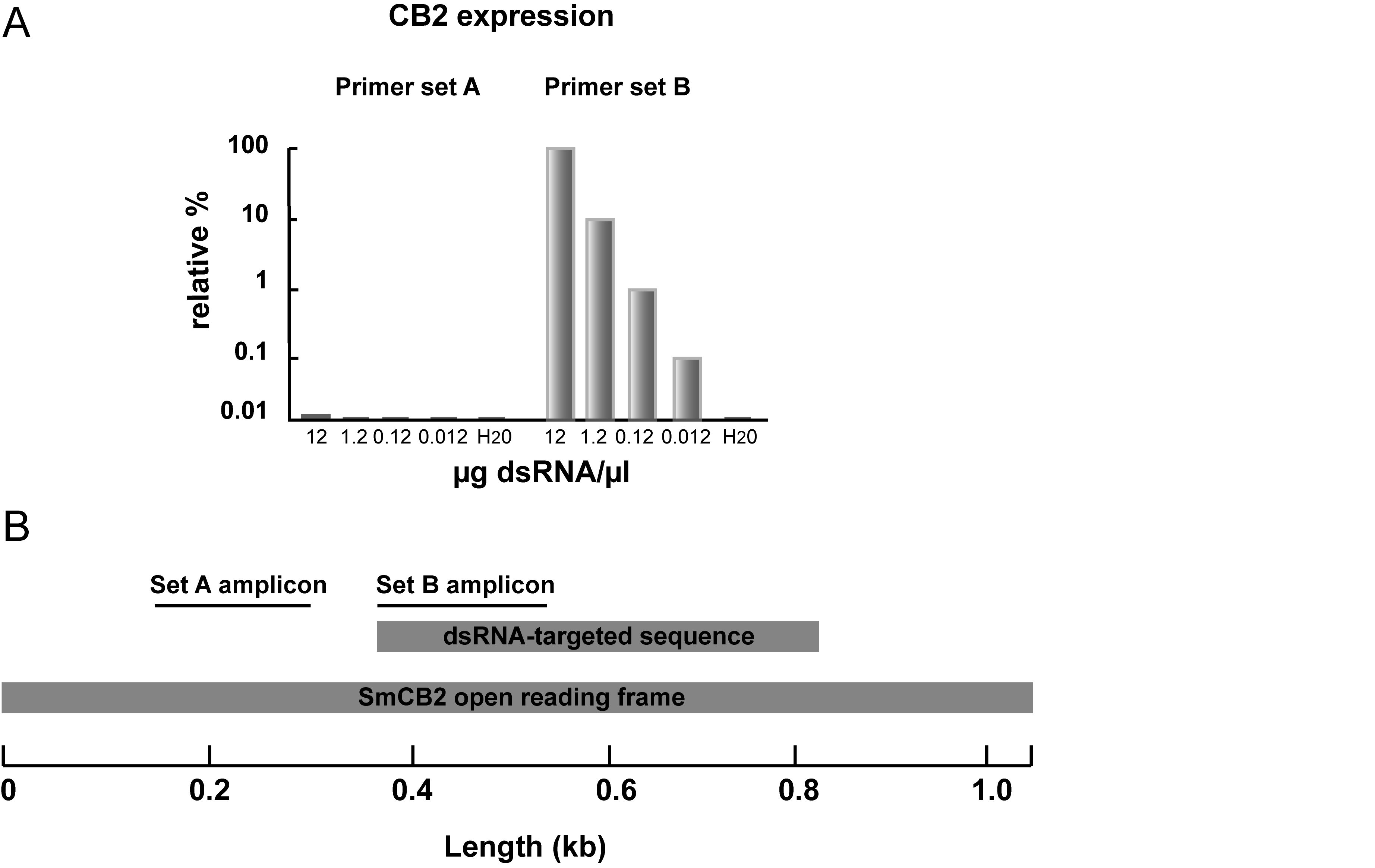

Supplement: Figure S1 — The influence of qPCR primer positioning on the amplification of residual PCR product carried over from dsRNA synthesis. There is no amplification of residual PCR product (Panel A) when primer pairs (Primer set A) are positioned upstream from that part of the open reading frame of SmCB2 used to synthesize dsRNA (Panel B). In contrast, Primer set B (positioned within the sequence used to generate dsRNA) amplifies residual PCR product in a concentration-dependent manner that would negatively impact the apparent efficiency of RNAi as measured by qRT-PCR. Each sample was tested in duplicate and representative data from two experiments are shown. (0.35 MB TIF) [file pntd.0000850.s001.tif]
